# T Cell Maturation Stage Prior to and During GMP Processing Informs on CAR T Cell Expansion in Patients

**DOI:** 10.3389/fimmu.2016.00648

**Published:** 2016-12-26

**Authors:** Yarne Klaver, Sabine C. L. van Steenbergen, Stefan Sleijfer, Reno Debets, Cor H. J. Lamers

**Affiliations:** ^1^Laboratory of Tumor Immunology, Department of Medical Oncology, Erasmus MC-Cancer Institute, Rotterdam, Netherlands; ^2^Department of Medical Oncology, Erasmus MC-Cancer Institute, Rotterdam, Netherlands

**Keywords:** renal cell cancer, chimeric antigen receptor, carboxy-anhydrase-IX, T cell, immune monitoring, T cell maturation, T cell expansion, T cell persistence

## Abstract

Autologous T cells were genetically modified to express a chimeric antigen receptor (CAR) directed toward carboxy-anhydrase-IX (CAIX) and used to treat patients with CAIX-positive metastatic renal cell carcinoma. In this study, we questioned whether the T cell maturation stage in the pre-infusion product affected CAIX CAR expression and function *in vitro* as well as *in vivo* CAR T cell numbers and expansion. During the 14 days expansion of CAR T cells prior to administration, we observed shifts from a predominant CD4 to a CD8 T cell phenotype and from a significant fraction of naïve to central effector T cells. Surface expression of the CAR was equally distributed among different T cell subsets and T cell maturation stages. During T cell culture days 14–18 (which covered patient treatment days 1–5), T cells demonstrated a decline in CAR expression level per cell irrespective of T cell maturation stage, although the proportion of CAR-positive T cells and CAR-mediated T cell effector functions remained similar for both CD4 and CD8 T cell populations. Notably, patients with a higher fraction of naïve CD8 T cells at baseline (prior to genetic modification) or central effector CD8 T cells at 2 weeks of CAR T cell culture demonstrated a higher fold expansion and absolute numbers of circulating CAR T cells at 1 month after start of therapy. We conclude that the T cell maturation stage prior to and during CAR T cell expansion culture is related to *in vivo* CAR T cell expansion.

## Introduction

Despite clinical successes in B-cell malignancies, adoptive transfer of T cells genetically modified with chimeric antigen receptors (CARs) or T cell receptors (TCRs) to treat solid tumors is challenged by limited patient responses (1). The efficacy of adoptive T cell therapy (in hematological malignancies) correlates with numbers and persistence of circulating modified T cells (2–5). Building on this notion, several approaches have been explored to improve persistence of genetically modified T cells *in vivo*. For example, the introduction in receptors of intracellular domains from the CD28 and/or CD137 co-stimulatory molecules has led to an increased CAR T cell persistence as well as expansion *in vivo*, and consequently in clinical responses (6, 7).

Furthermore, preclinical studies in mice and monkeys suggest that improved *in vivo* persistence and antitumor responses are obtained when T cells in early stages of differentiation (such as naïve or central memory cells) are used for genetic modification and treatment (8, 9). In fact, the differentiation state of CD8^+^ T cells appeared to be inversely related to their capacity to proliferate and persist (10, 11). We have previously generated CAR T cells directed against carboxy-anhydrase-IX (CAIX) and treated patients with CAIX-positive metastatic renal cell carcinoma (mRCC) (12). Here, we assessed T cell maturation stage prior to and during CAR T cell expansion cultures, analyzed whether the T cell maturation stage affects CAR expression and function *in vitro* as well as the *in vivo* properties of CAR T cells, in particular expansion potential and absolute numbers of circulating CAR T cells.

## Materials and Methods

### Patients and Treatment

Patients, diagnosed with CAIX-positive mRCC and for whom no standard treatment was available, were included in this phase-I trial. Patients were treated in three cohorts, and aimed to assess toxicity and to establish the maximum tolerated dose of the number of CAR T cells. Treatment schedule was previously presented (13) and was, in brief: in cohort 1, treatment consisted of intravenous administration of 2 × 10^7^ T-cells at day 1, 2 × 10^8^ T-cells at day 2, and 2 × 10^9^ T-cells at days 3–5 (treatment cycle 1) and days 17–19 (treatment cycle 2). Simultaneously, patients received twice daily subcutaneous injections of interleukin-2 (IL-2) at 5 × 10^5^ IU/m^2^ on days 1–10 and days 17–26. Because of liver toxicity (13), this schedule was changed in cohort 2, to a classic 3 × 3 dose-escalating phase I schedule starting at 1 × 10^8^ CAR T-cells per infusion and extending to 2, 4, 8, 16, 20, 25, and 30 × 10^8^ cells at subsequent dose levels, and applying a maximum of 10 T-cell infusions at days 1–5 and days 29–33 combined with sc IL-2 at 5 × 10^5^ IU/m^2^ twice daily at days 1–10 and days 29–38. In cohort 3, patients were treated as in cohort 2, but received an extra i.v. infusion of 5 mg of the anti-CAIX mAb G250, 3 days before start of each series of CAR T-cell infusions, in order to block CAIX antigen in the liver and leaving accessible CAIX antigen at RCC tumor sites (14–16).

For the analyses of CAR T cell persistence, only patients treated in cohorts 2 and 3 were assessed until day 29, as from day 29 eight out of nine patients received a second treatment cycle of CAR T cells. Since patients in cohort 1 received varying numbers of CAR T cells, and one patient already received a second cycle of CAR T cells on days 17–19, cohort 1 was not assessed in the persistence analyses.

Patients did not receive lympho-depleting pretreatment. The clinical protocol and amendments were approved by governmental regulatory authorities (Central committee on research involving Human Subjects) as well as the Erasmus MC institutional medical ethical review board. The clinical protocol (DDHK97-29/P00.0040C) adheres to the Declaration of Helsinki protocols. Patient characteristics are detailed elsewhere (13).

### T Cell Infusion Product and Post-Treatment Blood Sampling

Patient peripheral blood mononuclear cells (PBMC) from leukapheresis (*n* = 9) were activated at day 0 with soluble anti-CD3 mAb OKT3 (10 ng/mL) without IL-2. At days 2 and 3, T cells were retrovirally transduced with the CAIX CAR in the presence of 100 IU/ml IL-2. From days 4 to 18, T cells were expanded in medium supplemented with 360 IU/ml IL-2. Patients were treated with five daily infusions of “fresh” CAIX CAR T cells harvested from culture at days 14–18 (13, 17). We obtained blood samples at regular intervals before, during, and after treatment for direct flow cytometric (FCM) analysis and isolation and cryopreservation of PBMC in liquid nitrogen (18, 19).

### Flow Cytometry

Carboxy-anhydrase-IX CAR-positive T cells in cultures and blood samples were assessed by FCM using the anti-CAIX CAR idiotype mAb NuH82, as described in Ref. (18) and Figure S1 in Supplementary Material. The starting T cell product (PBMC from leukapheresis) and T cell cultures were analyzed for various lymphocyte subsets, a.o. CD4^+^ and CD8^+^ T cells and T cell maturation subsets using following markers: CD27, CD28, CD45RA, CD45RO, CD62L, and CCR7. The starting T cell product (PBMC from leukapheresis) is referred to the “baseline” sample or measurement in the rest of this study. In addition, T cell maturation subsets were analyzed for CAR-expression (Figure S1 in Supplementary Material) and CAR-mediated effector function in response to CAIX^+^ RCC cell line (SKRC-17 MW1-clone4) by means of upregulated expression of CD107. Information on antibodies and staining combinations used in multi-color FCM is specified in Table S1 in Supplementary Material. Samples were measured on the FACS Canto, and analyzed with FCS express v. 4.07 software (*De Novo* software). Gating strategy to determine the T cell maturation stage is demonstrated in Figures S1A,B in Supplementary Material.

### Statistical Analysis

Statistical analyses were performed using SPSS software (version 21) for Windows (IBM Corporation, IL, USA). Graphpad Prism v5.0 was used to prepare graphs.

## Results

### T Cell Phenotype

**Figure 1:**
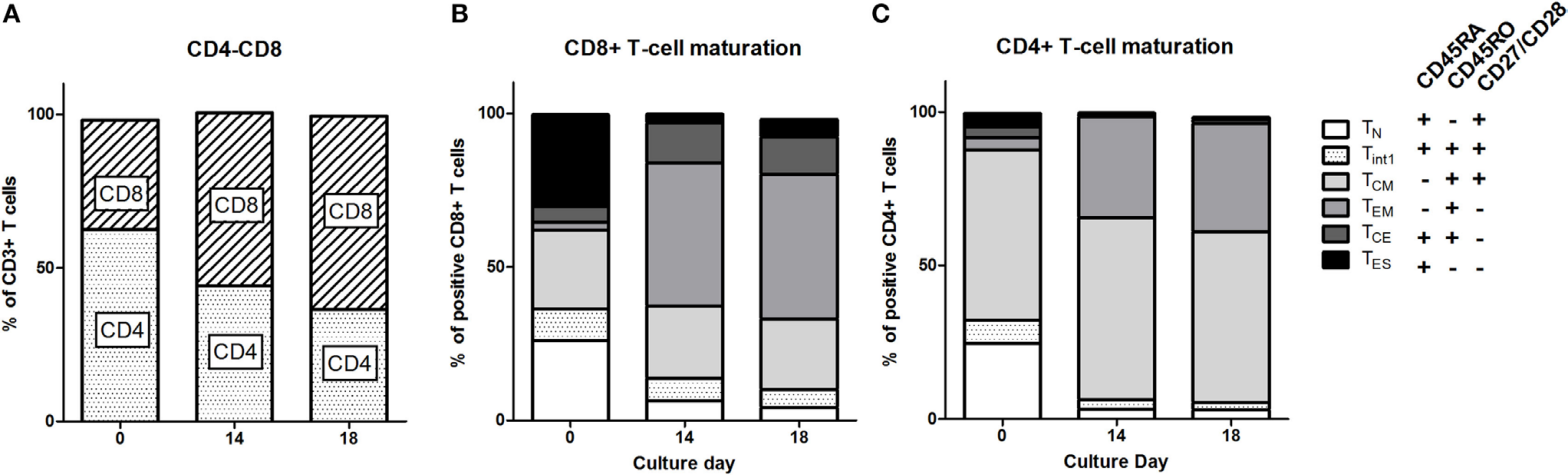
**T cell phenotype and maturation during clinical T cell expansion cultures**. **(A)** Proportions of CD4 and CD8 T cells at baseline (leukapheresis, day 0) and culture day 14. Data of individual patients are presented in Figure S1 in Supplementary Material. Maturation stages of CD8^+^ **(B)** and CD4^+^ **(C)** T cells at baseline and culture day 14 and at day 18 defined according to the expression of CD45RA, CD45RO, and CD27/CD28 as indicated in insert: Naïve, T_N_: CD45RA^+^, CD45RO^−^, CD27/CD28^+^; Central Memory, T_CM_: CD45RA^−^, CD45RO^+^, CD27/28^+^; Effector Memory, T_EM_: CD45RA^−^, CD45RO^+^, or CD27/CD28^−^; Central Effector, T_CE_: CD45RA^+^, CD45RO^+^, CD27/CD28^−^; and End Stage T_ES_: CD45RA^+^, CD45RO^−^, CD27/CD28^−^ T cells. A small fraction was CD45RA^+^, CD45RO^+^, and CD27/CD28^+^ and this population was defined as interphase (Int1) T cells. Data are presented as stacked bars of means of nine patients.

Patient PBMC at baseline (from leukapheresis) were activated, transduced with the CAIX CAR, and expanded. During the 14 days of expansion, the phenotype of the T cells shifted significantly. Although the extent varied per patient, T cell cultures reproducibly demonstrated a shift from a CD4^+^ to a CD8^+^ predominance compared to baseline (Figure 1A; Figure S2 in Supplementary Material). During the 18 days culture period, individual markers expressed on CD3^+^ T cells demonstrated a clear shift, especially during the first 14 days (Figure S3 in Supplementary Material). When assessing CD8 T cell maturation according to the markers CD45RA, CD45RO, CD27, and CD28, we observed that during culture the most prevalent subset drastically shifted from T_ES_ at baseline to T_EM_ at day 14 [Figure 1B; see legend of Figure 1 for the definition of maturation stages: T_N_ (naïve), T_INT_ (intermediate between T_N_ and T_CM_), T_CM_ (central memory), T_EM_ (effector memory), T_CE_ (central effector), and T_ES_ (end stage) T cells]. Following T cell culture, there was also a decrease in the fraction of T_N_ and an increase in the fraction of T_CE_, yet the overall change was in favor of younger T cells (T_CM_ + T_EM_) at the expense of further maturated T cells (T_CE_ + T_ES_). CD4^+^ T cells also showed a culture-dependent decrease in the fraction of T_N_ and an increase in the fraction of T_EM_, yet the most prevalent subset at baseline, i.e., T_CM_, remained unchanged (Figure 1C). Interestingly, in contrast to CD8^+^ T cells cultured CD4^+^ T cells harbored almost no T_CE_ cells. Additional analysis with different T cell maturation markers [CD45RA, CCR7 (CD197), and CD62L] showed high concordance with the maturation stages and kinetics as described above (Figure S4 in Supplementary Material).

### CAIX CAR Expression in T Cell Maturation Subsets

**Figure 2:**
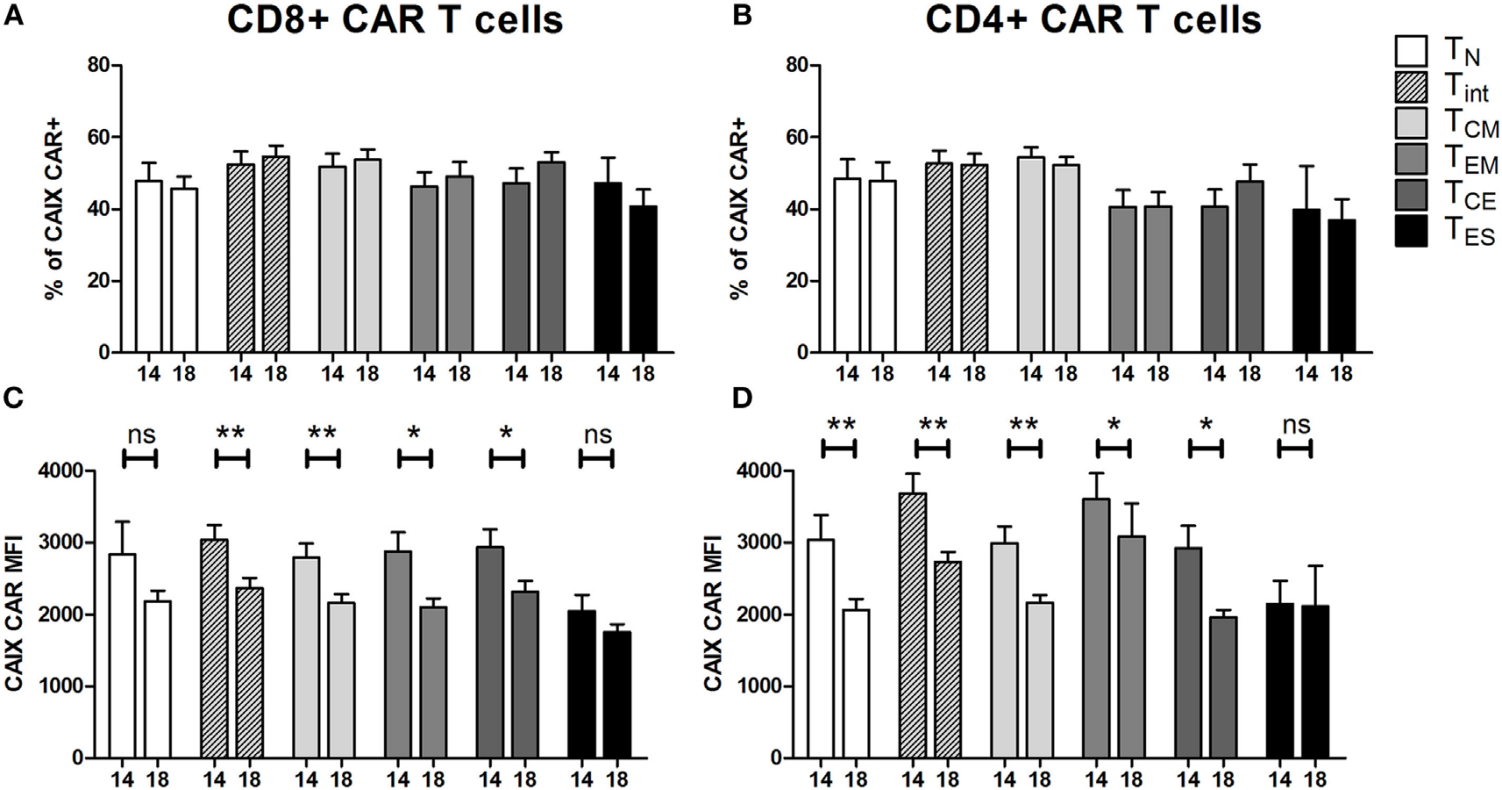
**Carboxy-anhydrase-IX (CAIX) chimeric antigen receptor (CAR) expression of T cells in infusion products according to T cell maturation**. Proportions of CAIX CAR-positive CD8^+^ **(A)** and CD4^+^ **(B)** T cells and CAIX CAR expression levels (expressed as mean fluorescence intensity) on CD8^+^ **(C)** and CD4^+^ **(D)** T cells at culture days 14 and 18 according to T cell maturation. Differences between culture days 14 and 18 with respect to paired continuous parameters were determined using an exact Wilcoxon rank-sum test. *p < 0.05; **p < 0.01; ns, not significant. Bars represent mean ± SEM from 16 independent clinical CAIX CAR T cell cultures for the treatment of nine patients, of which seven received infusions in two treatment cycles. See also legend to Figure 1.

**Figure 3:**
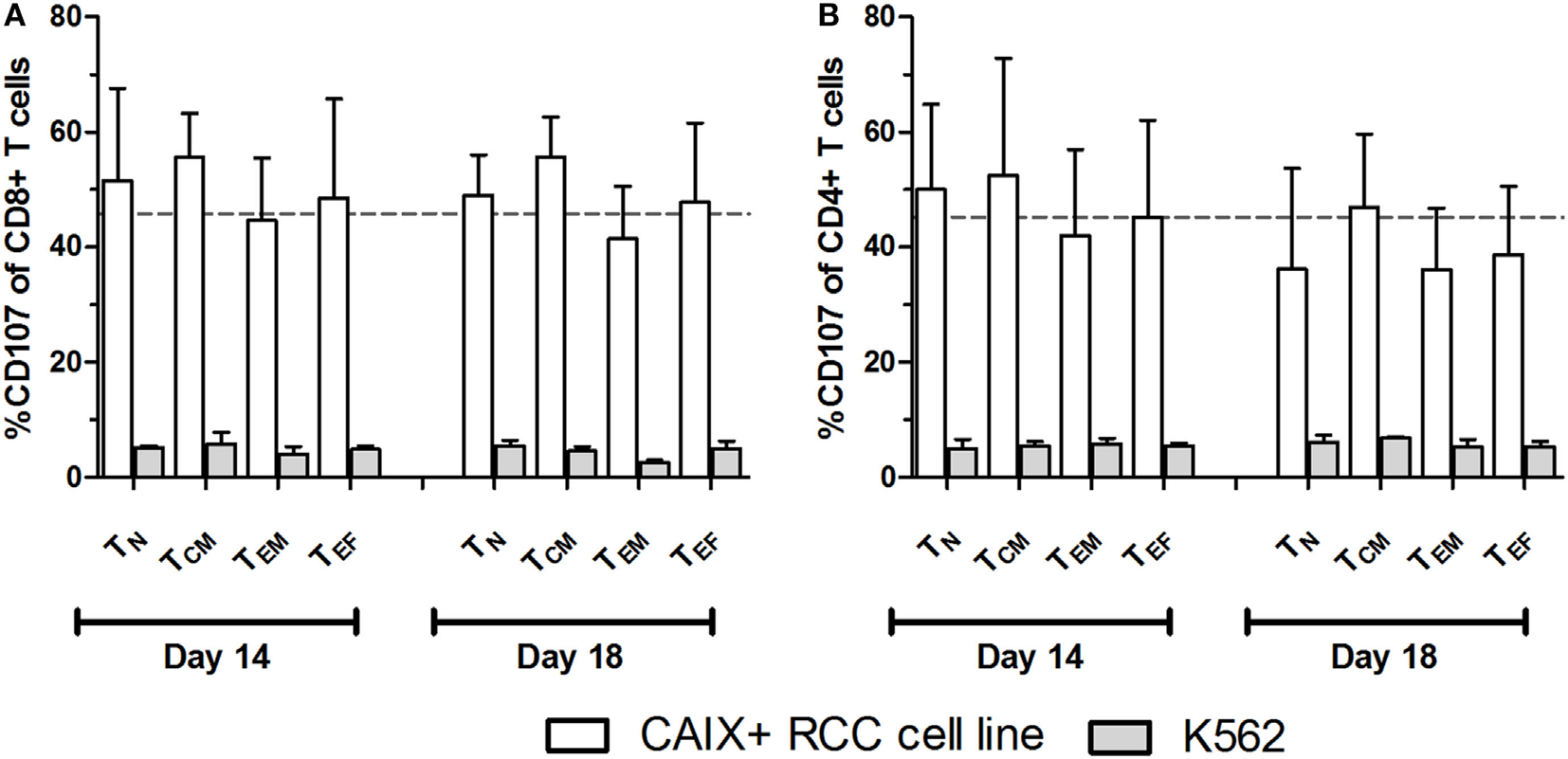
**Carboxy-anhydrase-IX (CAIX) chimeric antigen receptor (CAR)-mediated function by T cell maturation subsets**. CAIX CAR T cell cultures at days 14 and 18 were assayed for CAIX CAR-mediated cytolytic (degranulation) potential. CAIX CAR T cells were co-cultured (2 h) with the CAIX-positive RCC cell line (SKRC-17 MW1-clone4) or the CAIX-negative cell line K562 and subsequently analyzed by flow cytometry. Results are presented as proportions of CD107 positivity within differently matured CD8^+^ **(A)** and CD4^+^ **(B)** T cells. Dotted line represents the average proportion of CD107 positivity of CD8^+^ T cells after co-culture with the CAIX-positive RCC cell line. T cell maturations subsets were defined by the markers CD45RA and CCR7 as follows: T_N_: CD45RA^+^, CCR7^+^; T_CM_: CD45RA^−^, CCR7^+^; T_EM_: CD45RA^−^, CCR7^−^; T_EF_: CD45RA^+^, CCR7^−^. Bars represent mean ± SEM (*n* = 3).

At culture day 14, the proportion of CAIX CAR-expressing cells was about equal for various T cell subpopulations, such as CD3 positive T cells in combination with CD4, CD8, CD56, CD57, TCRγδ, CD45RA, CD45RO, CD62L, or CCR7 (Figure S5 in Supplementary Material). The proportion of CAIX CAR-expressing cells within CD8^+^ and CD4^+^ T cells was stable during patient treatment (culture days 14–18), and appeared highest in the T_INT_^+^ T_CM_ and lowest in the T_ES_ maturation stages (Figures 2A,B). When considering the CAR expression level per cell (mean fluorescence intensity, MFI), we observed a significant decrease of CAR expression between days 14 and 18 for almost all maturation stages with exception of CD8^+^ T_N_, CD8^+^ T_ES_ cells, and CD4^+^ T_ES_ cells (Figures 2C,D). This observation is in extension to a previous report on a general loss of CAR expression during the last 5 days of the T cell culture (20). Data at MFI level also reinforced the above observation that CAR expression was highest in the T_INT_ + T_CM_ and lowest in the T_ES_ maturation stages (Figures 2C,D). Further, we analyzed the CAIX CAR-mediated function (degranulation) and found no significant differences in CD107-upregulation between the different maturation stages or T cell culture times following co-culture of CAR T cells with a CAIX-positive RCC cell line or CAIX-negative K562 cells. Thus, the relatively small decrease in CAR expression did not result in a measurable decrease in CAR-mediated function (Figures 3A,B).

### T Cell Maturation Correlates with *In Vivo* CAR T Cell Expansion

**Figure 4:**
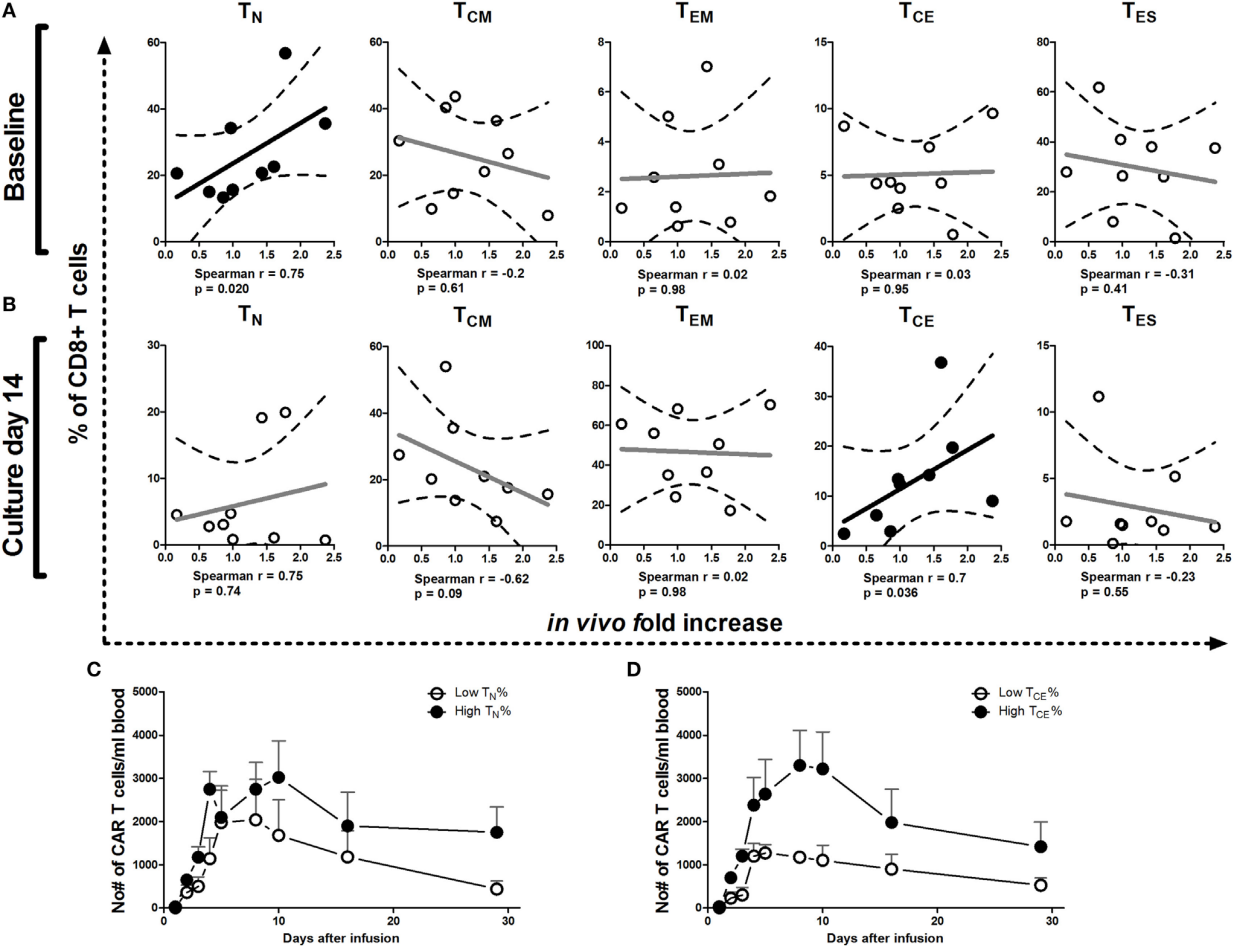
**Correlations between T cell maturation (pre-infusion) and increase of chimeric antigen receptor (CAR) T cell numbers in patients**. Correlation plots of proportions of T_N_, T_CM_, T_EM_, T_CE_, and T_ES_ cells at baseline **(A)** and culture day 14 **(B)** vs. increase of CAR T cell numbers *in vivo* during the first 5 days after the last T cell infusion of treatment cycle 1 (13, 20). Dotted lines represent the 95% confidence bands of the best fitted line. The Spearman correlation coefficient method was used to assess linear association. *p*-Values less than 0.05 were considered significant. Patients were divided into above or below median values (high and low, respectively) of T_N_ at baseline (day 0) **(C)** or T_CE_ cells at culture day 14 **(D)** and plotted for absolute numbers of circulating CAR T cells during treatment cycle 1 (days 1–29). See also legend to Figure 1 for further details.

We analyzed whether phenotype of T cells at baseline and after culture correlated with numbers of CAR T cells in patient blood and expansion (fold increase) of these CAR T cells during 5 days after the last infusion. Kinetics of circulating CAR T cell numbers irrespective of maturation stage in patients have been reported elsewhere (20). Here, we reveal a significant correlation between the fold increase in CAR T cell numbers in patients and the proportions of CD8^+^ T_N_ cells at baseline (Figure 4A) and CD8^+^ T_CE_ in the infusion product at culture day 14 (Figure 4B) and on day 18 [*r* = 0.683, *p* = 0.042 (data not shown)]. Patients with higher proportions of CD8^+^ T_N_ cells in the baseline PBMC and CD8^+^ T_CE_ in the infusion product, in general, had higher absolute numbers of circulating CAR T cells up to 29 days after the first infusion (Figures 4C,D). Our *in vitro* analyses considered both CD8^+^ and CD4^+^ T cells (Figures 1–3). Correlations between the relative occurrence of T cell maturation stages (prior to and during T cell processing) and numbers of T cells in post-treatment patient blood samples were assessed for both CD8^+^ and CD4^+^ T cells. Significant correlations, however, were found only for CD8^+^ T cells but not CD4^+^ T cells (data not shown). We found no correlations between proportion of CAR-positive T cells or CAR expression (MFI) in the infusion product and CAR T cell numbers or expansion in patients (data not shown).

## Discussion

Here, we document T cell phenotypic changes during IL-2-supported CAIX CAR T cell cultures in preparation of a clinical trial to treat RCC patients. During the 2-week expansion period, T cells skewed from a CD4 to CD8 phenotype and the proportion of naïve CAR T cells (T_N_: CD45RA^+^, CD45RO^−^, CD27/CD28^+^) strongly declined, while the proportions of T_EM_ (CD45RA^−^, CD45RO^+^, CD27/CD28^−^) and T_CE_ (CD45RA^+^, CD45RO^+^, CD27/CD28^−^) cells significantly increased. We noted a shift from T_N_ cells to more maturated stages as was described before for other transduced T cell products cultured with IL-2 (21). Interestingly, also the frequency of T_ES_ declined during the CAR T cell culture. We anticipate that the less maturated cells like T_CM_ and T_EM_ have proliferated faster, and thereby have overgrown the T_ES_ cells. We demonstrated a small decline in CAR expression level per cell during the 5-day CAR T cell culture covering the days of T cell administration (days 14–18) that was independent of T cell maturation stage and did not affect CAR-mediated function. We conclude that the five sequential and “freshly” prepared clinical preparations of CAIX CAR T cells had about equal phenotypic and functional properties.

In adoptive CAR T cell treatment, circulating numbers, persistence and *in vivo* expansion potential of infused CAR T cells are currently the only parameters revealed to be associated with improved clinical outcome (22). Most CAR T cell trials targeting CD19 in hematological malignancies show strong T cell expansion, mainly due to a high load and accessibility of target antigen, the nature of tumor cells (B cells being able to provide co-stimulation), and highly active (second generation) CAR T cells. Along these lines, we sought to define pre-infusion characteristics that may predict the *in vivo* behavior of infused CAR T cells. Here, we showed that patients with more CD8^+^ T_N_ cells in baseline PBMC (from leukapheresis) or more CD8^+^ T_CE_ in the infusion product at culture day 14, showed a higher fold increase in numbers of CAR T cells *in vivo*, resulting in higher blood levels of CAR T cells in these patients for at least 4 weeks. In fact, a correlation exists between characteristics of both baseline PBMC (proportion CD8^+^ T_N_) and infusion product (proportion CD8^+^ T_CE_) and numbers of circulation CAR T cells after treatment. Such findings bear clinical relevance, as younger T cells were shown to positively correlate with clinical effectiveness in adoptive T cell trials (1, 23, 24). To the best of our knowledge, our study is the first CAR T cell trial targeting a solid tumor, in which a correlation is demonstrated between pre-infusion and pre-expansion T cell characteristics and *in vivo* CAR T cell expansion potential. In only one other non-CD19 CAR trial, targeting neuroblastoma with GD2 CAR T cells, a correlation was described between *in vivo* CAR T cell persistence and the proportion of CD4^+^ or T_CM_ cells in the infusion product (24). Our observation is in line with the report that effector T cells derived from naive rather than memory T cell subsets possess superior features for adoptive immunotherapy, though no correlations were made with *in vivo* parameters (25).

The observation that the proportion CD8^+^ T_N_ in baseline PBMC determines the *in vivo* behavior of CAR T cells is intriguing and provides a means to manipulate leukapheresis products for a better clinical outcome, especially for patients with low proportions of T_N_ cells in PBMC. This could include enrichment for T_N_ cells before transduction or changing the culturing conditions by addition of cytokines such as IL-15 and IL-21 during expansion (21, 26). These data may provide clues to adapt the *in vitro* T cell processing toward optimal T cell fitness and may enable to develop an improved protocol for adoptive T cell therapy.

## Ethics Statement

The manuscript contains clinical study data. The clinical protocol and amendments were approved by governmental regulatory authorities (Central committee on research involving Human Subjects) as well as the Erasmus MC institutional medical ethical review board. The clinical protocol (DDHK97-29/P00.0040C) adheres to the declaration of Helsinki protocols.

## Author Contributions

YK contributed in conception and design of the study, the analysis, planned and directed the statistical analysis, was involved in interpretation of data, and drafted the manuscript. SCS was involved in acquisition of the data and assisted in drafting the manuscript. SS contributed to conception and design of the study, and assisted in revising of the manuscript. RD contributed in conception and design of the study, interpretation of data, and drafting and revising of the manuscript. CL contributed in conception and design of the study, analysis and interpretation of data, and assisted in drafting and revising the manuscript. All the authors read, critiqued, and approved the final manuscript. All the authors also agreed to be accountable for all aspects of the work.

## Conflict of Interest Statement

The authors declare that research was conducted in the absence of any commercial or financial relationships that could be construed as a potential conflict of interest.

## Abbreviations

CAIX, carboxy-anhydrase-IX; CAR, chimeric antigen receptor; CD, cluster of differentiation; mRCC, metastatic renal cell carcinoma; PBMC, peripheral blood mononuclear cells; IL-2, interleukin-2; FCM, flow cytometry; T_N_, naïve T cells; T_INT_, intermediate T cells; T_CM_, central memory T cells; T_EM_, effector memory T cells; T_CE_, central effector T cells; T_ES_, end stage T cells.
