# Supplementary material for: T Cell Maturation Stage Prior to and During GMP Processing Informs on CAR T Cell Expansion in Patients
Source: Front Immunol. 2016 Dec 26;7:648. doi: 10.3389/fimmu.2016.00648 (PMC5183620; doi:10.3389/fimmu.2016.00648)
Supplement: Supplementary file 1 [file data_sheet_1.pdf]

T cell maturation stage prior to and during GMP processing informs on CAR T cell expansion in patients.

Yarne Klaver[1], Sabine C.L. van Steenbergen[1], Stefan Sleijfer[2], Reno Debets[1] and Cor H.J. Lamers[1,*]

[1]Laboratory of Tumor Immunology, Department of Medical Oncology, Erasmus MC-Cancer Institute, Rotterdam, The Netherland;

[2]Department of Medical Oncology, Erasmus MC-Cancer Institute, Rotterdam, The Netherlands;

***Corresponding author:**
CHJ Lamers, PhD, Laboratory of Tumor Immunology, Department of Medical Oncology, Erasmus MC-Cancer Institute, room Be430C, PO Box 2040, 3000 CA Rotterdam, The Netherlands; Tel: +31-10-7041771; E-mail: c.lamers@erasmusmc.nl

Supplementary table S1:
Antibodies used in flow cytometry of T-cell cultures

Antibody	Fluorochrome	Clone	Company*
Tube 1: major lymphocyte subsets (1)			
CD3	APC	SK7	BD Biosciences
CD4	PE-Cy7	SK3	BD Biosciences
CD8	FITC	SK1	BD Biosciences
CD56	PE	C5.9	Dako Cytomation
7-AAD	(PerCP)		Sigma-Aldrich
NuH82-biotin	--		Custom biotinylated
Streptavidin	APC-Cy7		BD Biosciences
Tube 2: major lymphocyte subsets (2)			
CD3	APC	SK7	BD Biosciences
CD4	PE-Cy7	SK3	BD Biosciences
CD8	PerCP	SK1	BD Biosciences
CD57	FITC	HNK-1	BD Biosciences
TCRγδ	PE	11F2	BD Biosciences
NuH82-biotin	--		Custom biotinylated
Streptavidin	APC-Cy7		BD Biosciences
Tube 3: T lymphocyte maturation (1)			
CD3	APC	SK7	BD Biosciences
CD8	PerCP	SK1	BD Biosciences
CD27	PE	M-T271	BD Biosciences
CD28	PE	CD28.2	BD Biosciences
CD45RA	FITC	L48	BD Biosciences
CD45RO	PE-Cy7	UCHL-1	BD Biosciences
NuH82-biotin	--		Custom biotinylated
Streptavidin	APC-Cy7		BD Biosciences
Tube 4: T lymphocyte maturation (2)			
CD3	APC	SK7	BD Biosciences
CD8	PerCP	SK1	BD Biosciences
CD62L	PE	SK11	BD Biosciences
CD45RA	FITC	L48	BD Biosciences
CCR7	PE-Cy7	3D12	BD Biosciences
NuH82-biotin	--		Custom biotinylated
Streptavidin	APC-Cy7		BD Biosciences
Tube 5: CD107 assay			
CD3	APC	SK7	BD Biosciences
CD8	PerCP	SK1	BD Biosciences
CD45RA	FITC	L48	BD Biosciences
CCR7	PE-Cy7	3D12	BD Biosciences
CD107a	PE	H4A3	BD Biosciences
NuH82-biotin	--		Custom biotinylated
Streptavidin	APC-Cy7		BD Biosciences

* BD Biosciences, San Jose, CA, USA; Dako Cytomation, Glostrup, Denmark; Sigma-Aldrich, St Louis, MO, USA.

Supplementary figure S1

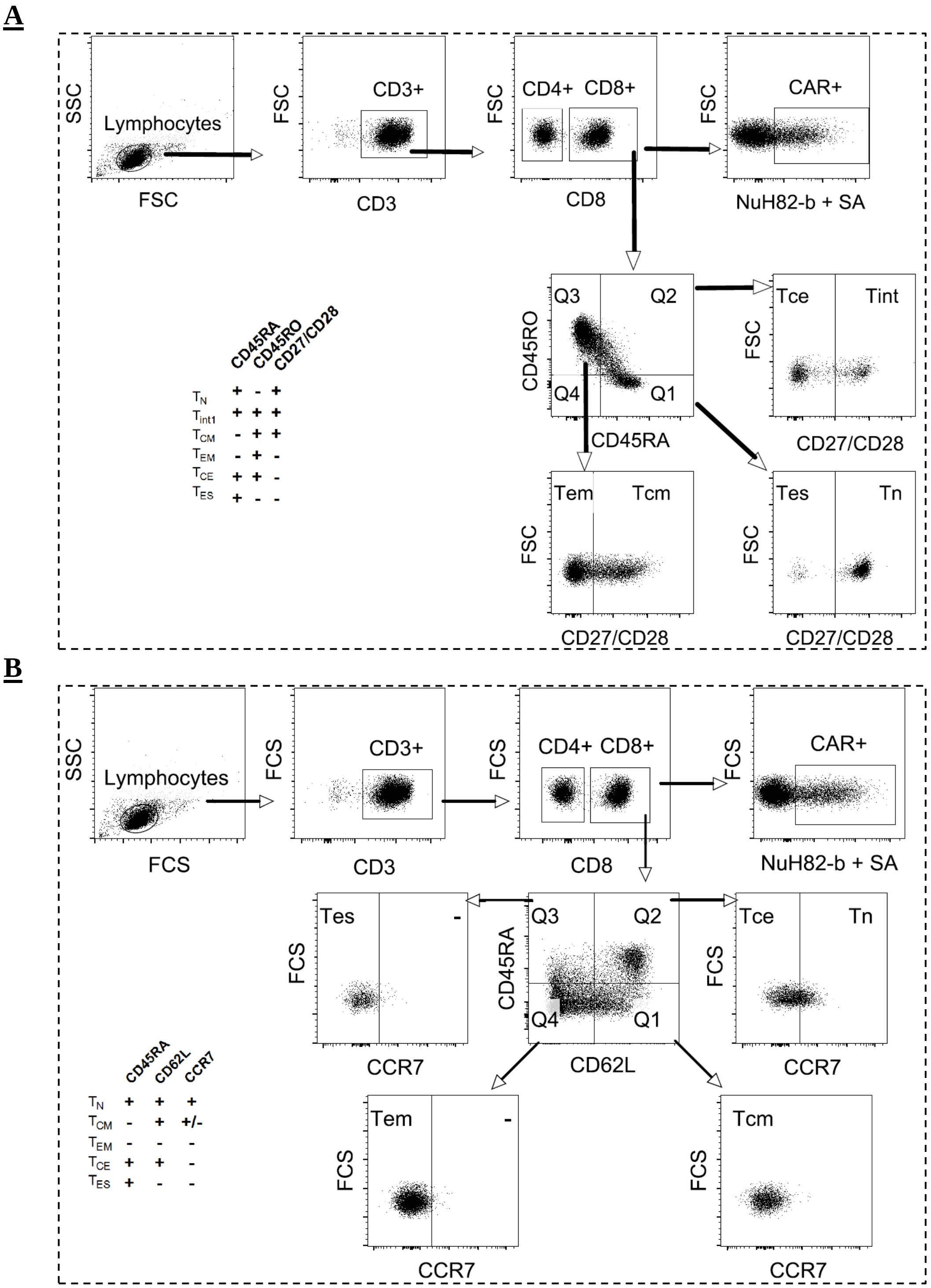

Supplementary figure S1: Gating strategy of T cell cultures for CAR expression and T cell maturation Gating strategy for the CD45RA/CD45RO/CD27/CD28 maturation panel, and (B) the gating strategy for the CD45RA/CD62L/CCR7 maturation panel. Both panels also determined the CAIX CAR expression (upper right panels). The NuH82-biotin (b) + Streptavidin (SA) gate was set based on an "unstained" tube, in which the NuH82-biotin mAb was left out in the first staining step. A Boolean gating approach, was used to automatically generate the combinations of the maturation stage and CAIX CAR expression. Plots show a representative T cell culture at day 14 of the culture.

Supplementary figure S2

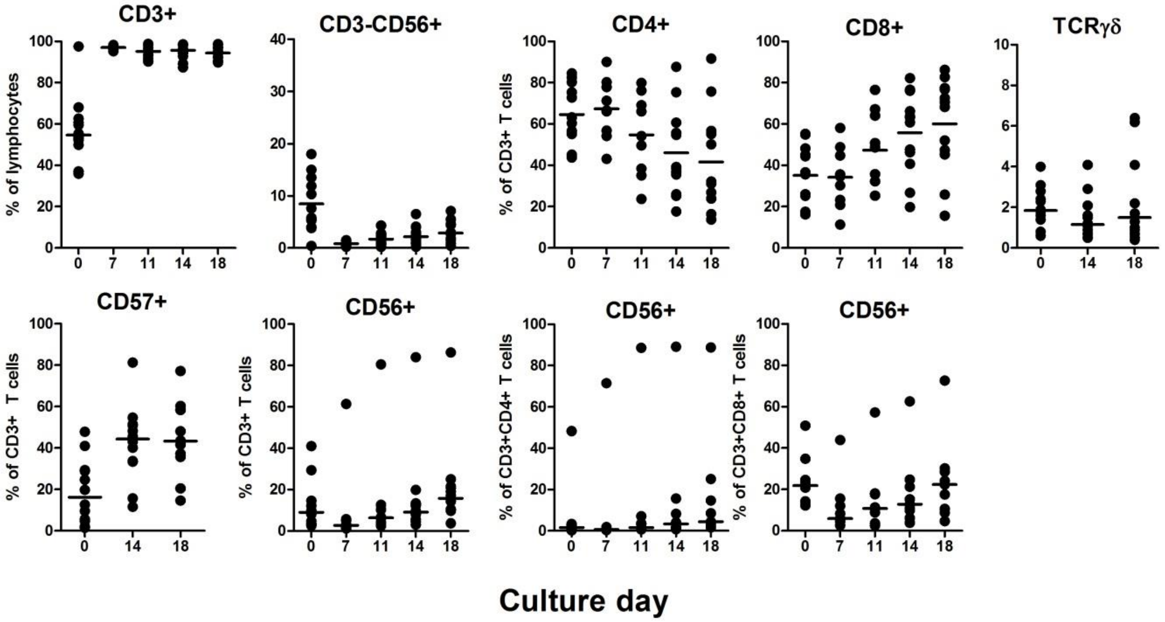

Supplementary figure S2: Kinetics of major lymphocyte subsets during CAIX CAR T-cell expansion cultures. Patient PBMC from 9 patients were activated, transduced and cultured two-times as detailed in Materials and Methods section to generate therapeutic doses of CAIX CAR T-cells for treatment cycle 1 and treatment cycle 2; for both clinical cultures, non-transduced T-cells were cultured in parallel, resulting in 4 independent T-cell cultures per patient. FCM analysis of lymphocyte subsets revealed that phenotypic shifts were independent of transduction and reproducible for each patient, therefore mean values of the 4 individual cultures are presented (one dot per patient per time point). Day 14 and day 18, represent the infusion product on the first and last of the 5 daily infusions of CAIX CAR T-cells. Presented lymphocyte subsets: $CD3^+$; $CD3^-CD56^+$ (NK); $CD3^+CD4^+$; $CD3^+CD8^+$; $CD3^+TCRgd^+$; $CD3^+CD57^+$; $CD3^+CD56^+$, and % $CD56^+$ of $CD3^+CD4^+$, and % $CD56^+$ of $CDC3^+CD8^+$. Horizontal lines represent the median values.

Supplementary figure S3

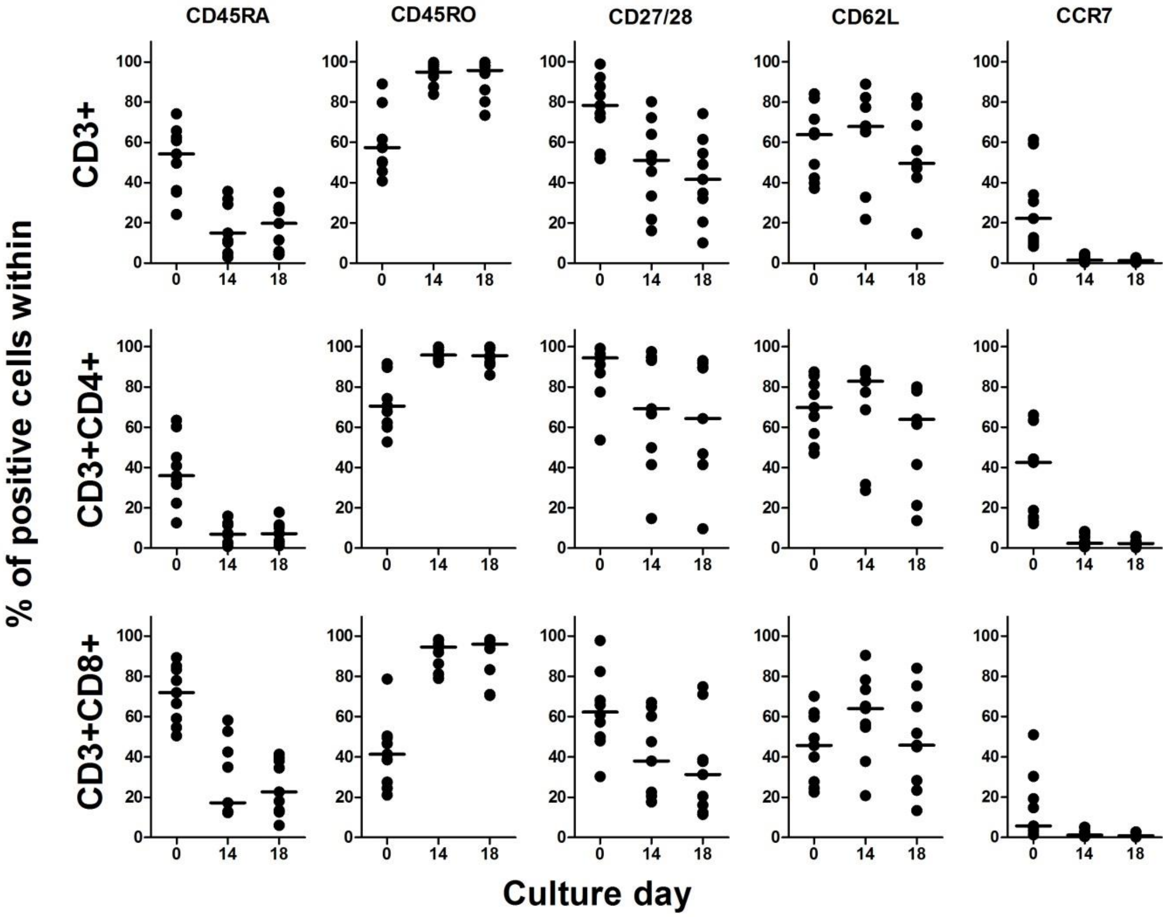

Supplementary figure S3: Kinetics of T-cell maturation markers expressed by different T-cell subsets during CAIX CAR T-cell expansion cultures. Patient PBMC from 9 patients were propagated as detailed in the legend to Figure S2. FCM analysis of maturation markers CD45RA, CD45RA, CD27/CD28, CD62L and CCR7 on $CD3^+$ T-cells and $CD3^+CD4^+$ and $CD3^+CD8^+$ T-cells was performed at baseline and culture days 14 and 18. The mean values of the 2 clinical CAIX CAR T-cell cultures are presented (one dot per patient /time point). Horizontal lines represent the median values.

Supplementary figure S4

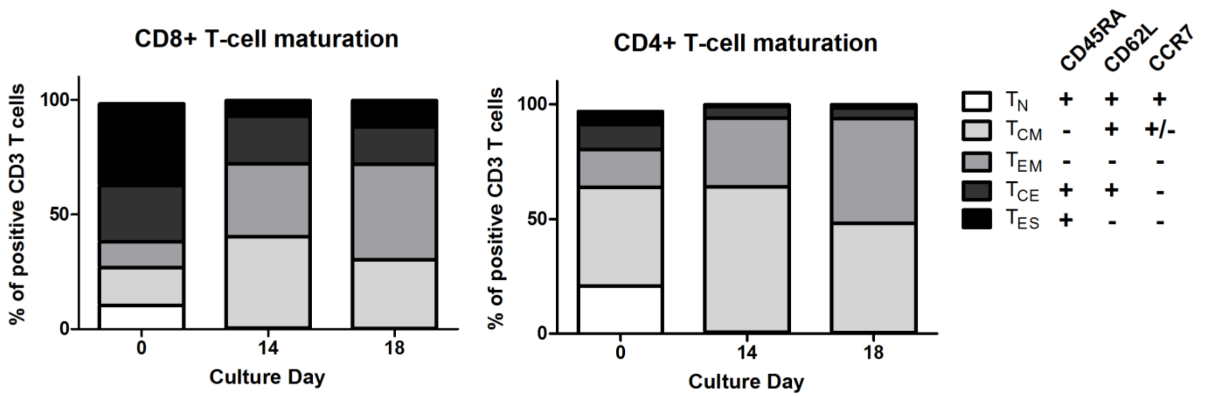

Supplementary figure S4: T-cell maturation during clinical T-cell expansion cultures using CD45RA, CD62L and CCR7. CAIX CAR T-cell cultures were generated as described. Proportions of T-cell maturation subsets within CD8+ (A) and CD4+ (B) T-cells were assessed at baseline (PBMC from leukapheresis, day 0), culture day 14, and culture day 18 according to the expression of CD45RA, CD62L and CCR7 as indicated the in insert: Naïve, T_N: $CD45RA^+$, $CD62L^+$, $CCR7^+$; Central Memory, T_{CM}: CD45RA-, $CD62L^+$, $CCR7^{+/-}$; Effector Memory, T_{EM}: $CD45RA^-$, $CD62L^-$, $CCR7^-$; Central Effector, T_{CE}: $CD45RA^+$, $CD62L^+$, $CCR7^-$; and End Stage T_{ES}: $CD45RA^+$, $CD62L^-$, $CCR7^-$; T-cells. Data are presented as stacked bars of means of 9 patients.

Supplementary figure S5

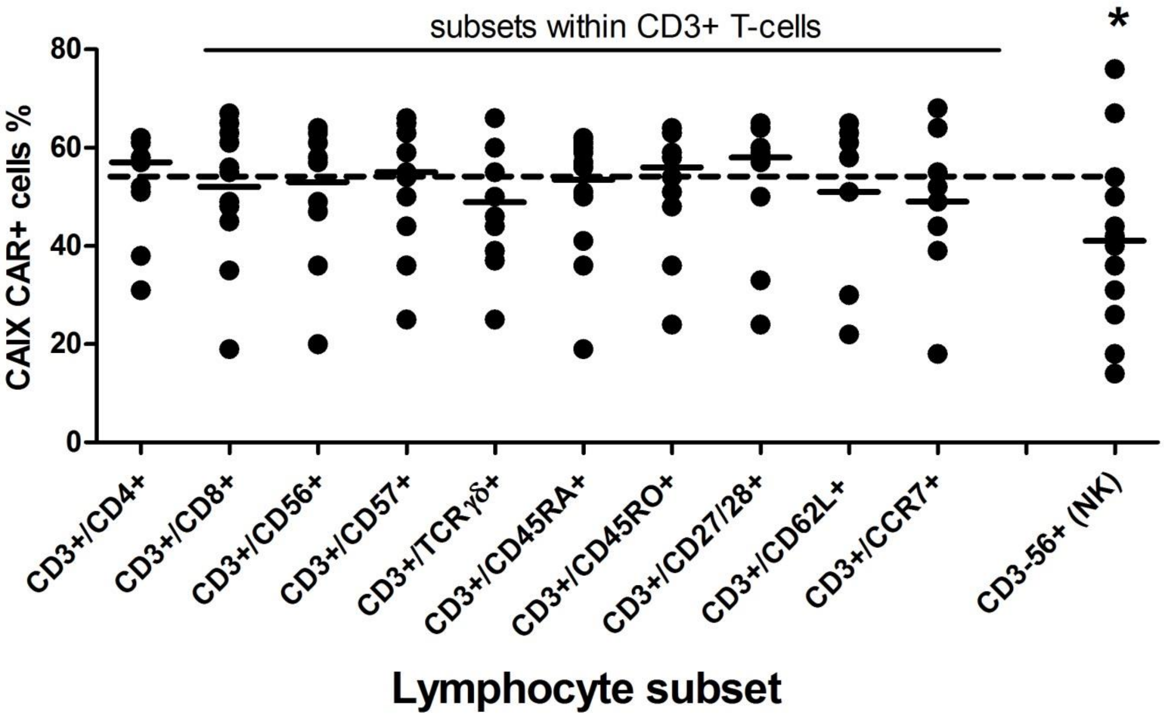

Supplementary figure S5: CAIX CAR is expressed homogenously among different T-cell subsets. CAIX CAR T-cell cultures were generated as described. At culture day 14 proportions of CAIX CAR$^+$ cells were assessed per lymphocyte subset by FCM. The mean values of CAIX CAR expression of the 2 individual CAIX CAR T-cell cultures are presented (one dot per patient). Lymphocyte subsets inlcuded: CD3$^+$; CD3$^+$CD4$^+$; CD3$^+$CD8$^+$; CD3$^+$CD56$^+$; CD3$^+$CD57$^+$; CD3$^+$TCRgd$^+$; CD3$^+$CD45RA$^+$; CD3$^+$CD45RO$^+$; CD3$^+$CD27/28$^+$; CD3$^+$CD62L$^+$; CD3$^+$CCR7$^+$, and CD3-CD56$^+$ (NK) cells. Graphs represent individual (dot) and median values (horizontal line). Differences from expression in CD3$^+$ were tested using the paired Student t-test, * $p < 0.05$.
